# Research on Recognition of Coal and Gangue Based on Laser Speckle Images

**DOI:** 10.3390/s23229113

**Published:** 2023-11-11

**Authors:** Hequn Li, Qiong Wang, Ling Ling, Ziqi Lv, Yun Liu, Mingxing Jiao

**Affiliations:** 1 School of Mechanical and Precision Instrument Engineering, Xi’an University of Technology, Xi’an 710048, China; wq1753456981@163.com (Q.W.); zero6927@163.com (L.L.); lyun@xaut.edu.cn (Y.L.); jiaomx@xaut.edu.cn (M.J.); 2 School of Chemical and Environmental Engineering, China University of Mining & Technology-Beijing, Beijing 100083, China; lzq_cumtb@hotmail.com

**Keywords:** coal gangue recognition, laser speckle, gray feature, texture feature, illuminance

## Abstract

Coal gangue image recognition is a critical technology for achieving automatic separation in coal processing, characterized by its rapid, environmentally friendly, and energy-saving nature. However, the response characteristics of coal and gangue vary greatly under different illuminance conditions, which poses challenges to the stability of feature extraction and recognition, especially when strict illuminance requirements are necessary. This leads to fluctuating coal gangue recognition accuracy in industrial environments. To address these issues and improve the accuracy and stability of image recognition under variable illuminance conditions, we propose a novel coal gangue recognition method based on laser speckle images. Firstly, we studied the inter-class separability and intra-class compactness of the collected laser speckle images of coal and gangue by extracting gray and texture features from the laser speckle images, and analyzed the performance of laser speckle images in representing the differences between coal and gangue minerals. Subsequently, coal gangue recognition was achieved using an SVM classifier based on the extracted features from the laser speckle images. The fusion feature approach achieved a recognition accuracy of 94.4%, providing further evidence of the feasibility of this method. Lastly, we conducted a comparative experiment between natural images and laser speckle images for coal gangue recognition using the same features. The average accuracy of coal gangue laser speckle image recognition under various lighting conditions is 96.7%, with a standard deviation of the recognition accuracy of 1.7%. This significantly surpasses the recognition accuracy obtained from natural coal and gangue images. The results showed that the proposed laser speckle image features can facilitate more stable coal gangue recognition with illumination factors, providing a new, reliable method for achieving accurate classification of coal and gangue in the industrial environment of mines.

## 1. Introduction

Coal is an important primary energy that plays a significant role in supporting the steady and rapid development of the global economy and society. During the process of coal mining, separation coal from gangue (the waste mineral) represents a critical processing step, promoting the efficient and comprehensive utilization of coal. Traditional methods for coal and gangue separation involve manual sorting, where operators rely on their subjective judgment based on the appearance, density, and other characteristics of coal and gangue for identification and separation. This method heavily relies on the subjective judgment of operators, which introduces a level of variability and potential errors in the separation process. Moreover, manual sorting is labor-intensive and time-consuming, resulting in increased costs and reduced efficiency. Mechanical separation techniques, such as the jigging process and heavy medium separation, are also used in industrial production [1,2]. These methods involve the efficient separation of coal and gangue through mechanical devices and physical principles. They are suitable for large-scale coal processing and mining production, meeting the requirements of high output and fast processing. However, mechanical separation techniques typically involve the use of heavy machinery, which can be costly to install, operate, and maintain. Additionally, the operation of such equipment can lead to environmental concerns, including noise pollution, water contamination, and potential environmental damage [3]. These issues indicate obvious shortcomings and limitations in traditional coal gangue separation technology.

With the rapid development of sensing and computer technologies, exploring more intelligent and automated technical solutions for coal gangue separation has become a hot topic in the field of coal mining [4,5]. In recent years, many scholars have conducted extensive research on coal gangue sorting based on image recognition technology [6,7,8,9,10,11]. Early image recognition methods were based on differences in grayscale and the texture of the surface natural images of coal and gangue, which were collected using CCD cameras. These methods utilized thresholds to distinguish coal and gangue by extracting statistical information about grayscale and texture as image features, achieving high accuracy in coal gangue recognition in laboratory settings [12,13,14,15,16,17]. However, it has been gradually found to be difficult to generalize these methods in practical industrial production, partly due to the stability issues associated with these surface image features. Wang et al. [18] investigated the impact of illumination levels in the image capture environment on the accuracy of coal gangue recognition in natural images. Further research into image recognition revealed that the natural image’s feature differences between coal and gangue undergo significant changes with the variation in illumination, and the pattern of these changes also shows marked differences between the two. Li et al. [19] extracted four grayscale parameters and four texture parameters of coal and gangue natural pictures collected under different illuminance conditions in the range of 2000–7500 lx. Through the normalization and comparison of these parameters, the influence of external light factors on the natural image features of coal and gangue was revealed. These studies indicate that natural image features are highly sensitive to changes in lighting conditions. Consequently, frequent retraining and adjustment of the recognition model are required in different mining sites or varying environmental lighting to ensure accurate coal gangue recognition. This significantly increases the complexity and cost of implementing coal gangue recognition in the field, resulting in reduced system stability in practical applications.

To improve the reliability of feature extraction while accounting for illumination, an increasing number of advanced sorting methods using different light sources have been proposed and tested by some scholars for automatic or intelligent recognition of coal and gangue, including infrared detection [20,21], X-ray detection [22,23], laser scanning [24], spectroscopic detection [25,26], and others. These technologies have improved some of the problems associated with current image recognition methods to varying degrees, but new shortcomings also exist. While infrared detection methods can address the influence of lighting conditions on image features, they are highly sensitive to environmental temperature and humidity, which may lead to inaccuracies in identification. X-ray detection methods have been widely applied in coal and gangue identification, but they require the use of radiation sources, which may pose radiation safety concerns. Laser scanning methods involve the emission of laser beams from a laser device to scan and measure coal and gangue, extracting shape and surface features for rapid identification. However, laser scanning methods are sensitive to factors such as occlusion, scattering, and reflection, which can introduce noise and incompleteness in the data, requiring complex algorithms for data processing and restoration. Spectral detection methods utilize the reflective and absorption characteristics of coal and gangue in different wavelength bands for identification, offering significant advantages in distinguishing coal and gangue with significant compositional differences. However, spectral detection equipment is costly and not suitable for large-scale applications. To enhance the level of coal and gangue image recognition in practical coal mining and processing operations, further research and technological improvements are needed to overcome the limitations of these methods and achieve more accurate, reliable, and economically viable coal and gangue identification techniques.

Since the advent of the laser technique, speckle generated by coherent lasers has received great attention. Many scholars have studied the statistical characteristics of speckle patterns and proposed techniques for measuring surface roughness [27,28,29,30,31,32]. Due to the differences in physicochemical structures between coal and gangue, significant differences exist in the roughness and reflective properties of their mineral surfaces [33]. Laser speckle images capture abundant information on the surface characteristics of coal and gangue minerals. Compared to other light sources, lasers possess strong anti-interference, directivity, and coherence. Thus, laser speckle images are less affected by collected illumination factors under complex industrial conditions, and the obtained image information is more reliable, without the radiation hazards. Therefore, using laser speckle imaging to identify coal and gangue has great potential.

This article proposes a novel method for coal gangue recognition based on laser speckle imaging and explores its feasibility and stability for distinguishing coal from gangue. Firstly, the principle of laser speckle was analyzed, and specific techniques for feature extraction, including grayscale and texture analysis, were introduced. The inter-class separableness and the intra-class compactness of collected laser speckle images of coal and gangue were evaluated using boxplots of the extracted features. The feasibility of this approach was confirmed by utilizing an SVM classifier based on the extracted features from coal gangue laser speckle images to recognize coal and gangue. Furthermore, to assess the stability of the proposed method in coal gangue recognition under varying illuminance levels, comparative experiments were conducted with a natural image-based feature recognition method. These experiments aimed to simulate the weak and unstable lighting conditions typically encountered in production site scenarios. Six sets of coal gangue natural images and coal gangue laser speckle images were collected at illuminance levels of 100, 288, 442, 672, 770, and 912 lux. The coal gangue recognition accuracy of these two types of images was compared under different illuminance levels to evaluate the stability of this new approach with respect to lighting conditions during image acquisition.

## 2. Methods

### 2.1. Laser Speckle Theory and Surface Characteristic Analysis of Coal Gangue

**Figure 1:**
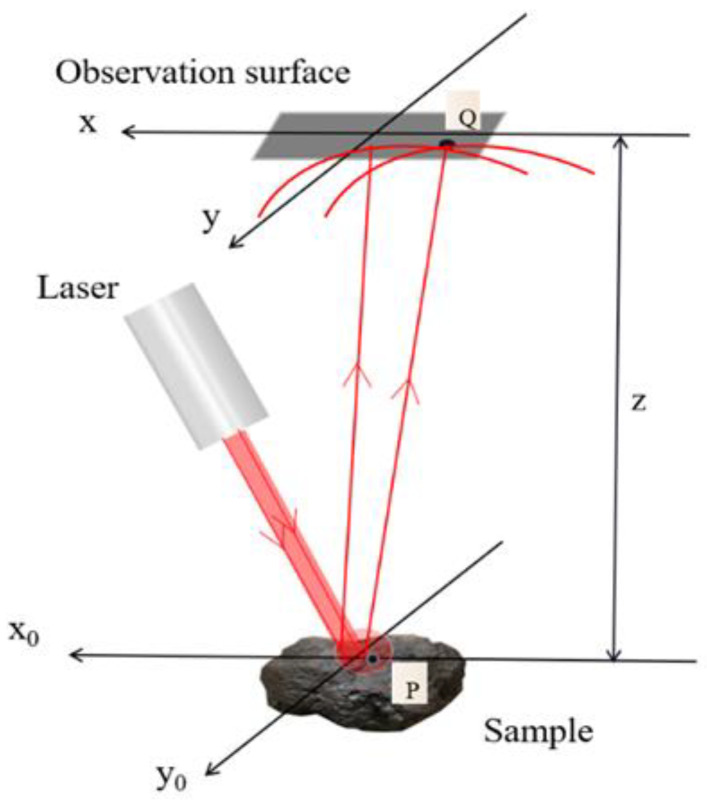
Principle of laser speckle imaging.

The molecular orientation, density fluctuation and interaction with matter in the surface medium cause light to deviate from the original propagation direction as the surface of coal and gangue is irradiated by single-wavelength light, which is light scattering. When coherent light is incident on the surface of a scattering medium, the scattering direction and intensity of light are influenced by factors such as particle size, shape, composition, and surface roughness, resulting in random scattering patterns. The mutual interference of these scattered light waves gives rise to a random distribution of speckles, known as laser speckle [34]. The observed scattering pattern in laser speckle carries detailed information about the surface roughness and morphology of the scattering medium, providing valuable insights into the unique characteristics of coal and gangue particles. The laser speckle imaging principle is shown in Figure 1.

The microscopic surface of coal and gangue is composed of a large number of mutually unrelated scattering points, and each point area is a diffraction surface element. When the laser beam is irradiated to the diffraction surface element point, a complex amplitude superposition is formed on the observation plane. According to the Fresnel principle [35], the complex amplitude of a point on the observation plane is

(1) $$U(Q)=\iint_{\varepsilon }U_{0}(P)h(P,Q)ds,$$

where ε is the coherent illumination region, and for any point *P* on the ε plane, its complex amplitude is *U*_0_(*P*), and s is a diffraction surface element. h(P,Q) is the impulse response from the diffraction plane to the observation plane.

If the aperture plane is in the *x*_0_*y*_0_ plane and the observation point is in the *xy* plane, Equation (1) can be expressed as follows:

(2) $$U(x,y)=\int \int_{-\infty }^{\infty }U_{0}(x_{0},y_{0})h(x_{0},y_{0};x,y)dx_{0}y_{0}$$

(3) $$h(x_{0},y_{0};x,y)=\frac{exp(jkz)}{j\lambda z}exp\left\{j\frac{k}{2z}\left[{\left(x-x_{0}\right)}^{2}+{\left(y-y_{0}\right)}^{2}\right]\right\}$$

With Fresnel approximation, the impulse response can be approximately expressed as:

(4) $$U(x,y)=\frac{exp(jkz)}{j\lambda z}\int \int_{-\infty }^{\infty }U_{0}(x_{0},y_{0})exp\left\{j\frac{k}{2z}\left[{\left(x-x_{0}\right)}^{2}+{\left(y-y_{0}\right)}^{2}\right]\right\}dx_{0}y_{0}$$

*z* is the distance from the diffraction surface element to the observation plane. The imaging system from the diffraction plane element to the observation plane is regarded as a linear space-invariant system. The light intensity at a point on the observation plane is expressed as:

(5) $$I(Q)=|U(Q){|}^{2}$$

It can be seen from the above that the speckle intensity distribution at each point of the observation plane is related to the distance *z* from the diffraction surface element to the observation plane, and the distance *z* is affected by the surface roughness. The speckle distributions of laser speckle images formed by different surface roughness of samples are also different.

**Figure 2:**
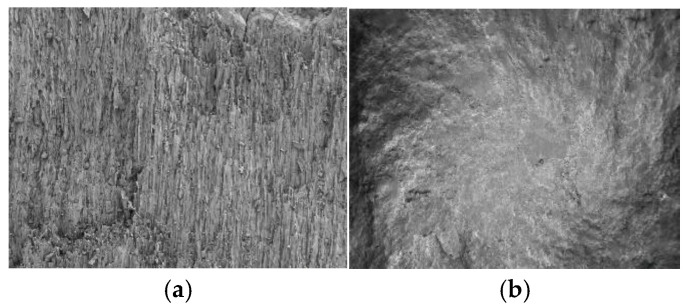
Microscopic surfaces of coal and gangue. (**a**) Coal; (**b**) gangue.

Coal and gangue have different surface morphology characteristics, and the surface microscopic diagram is shown in Figure 2. Coal is a porous rock with a rough and glossy surface [36,37], the molecular structure of the micro-surface is large, and the molecular accumulation is relatively loose under the scanning electron microscope image, as shown in Figure 2a. However, the gangue, with a glossy and dim surface, has a stable spatial structure, minerals are closely intergrown, the micro-surface is fine-grained, and it shows a flat structure under the scanning electron microscope image, as shown in Figure 2b.

**Figure 3:**
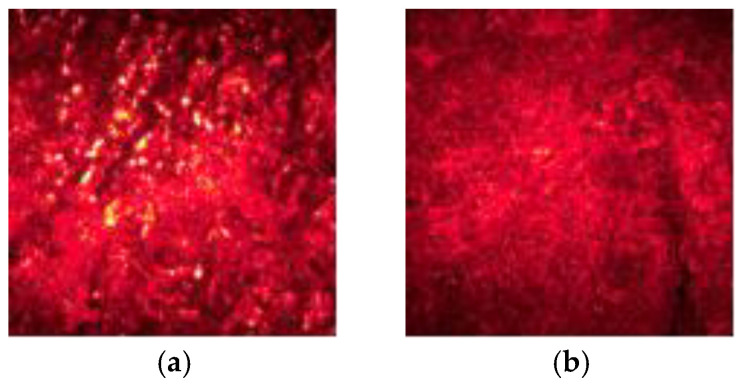
Laser speckle image. (**a**) Coal; (**b**) gangue.

The laser speckle image of coal gangue is formed by irradiating the surface of coal and gangue with coherent light, as shown in Figure 3. Due to the large size and loose arrangement of the molecular structure of the coal, the surface of coal is rougher, and the surface profile is more strongly scattered by coherent light, so, as shown in Figure 3a, the laser speckle contrast increases, the edge of the speckle intensity distribution is sharp, and the distribution of the flare is more obvious. The surface of the gangue is flat, with more fine particles, and the surface height fluctuation is small. In Figure 3b, the laser speckle images of the gangue show dense distribution and fewer bright spots. The brightness of its image is weaker than that of the coal speckle image. The statistical parameters of laser speckle images are used to quantitatively evaluate the distribution of light intensity in speckle imaging. Based on the statistical law of light intensity change, the gray histogram is used to extract the gray features of speckle images, and the texture features are extracted by typical texture feature methods, such as the gray level co-occurrence matrix [17,38], wavelet transform [39], Tamura [40], fractal dimension [41], etc. According to a large number of previous experiments, the gray level co-occurrence matrix has better performance in describing the texture change of coal gangue laser speckle.

### 2.2. Feature Extraction

#### 2.2.1. Grayscale Features

The gray histogram [11] of the image describes the overall statistical characteristics of the gray level distribution, namely the speckle distribution characteristics in the coal gangue laser speckle image. The gray histogram is expressed as Equation (6):

(6) $$p(b)=\frac{n(b)}{M\times N}$$

*b* is a gray level in a coal gangue laser speckle image, ranging from 0 to 255; *n(b)* represents the number of pixels of gray level b; *M × N* is the pixel area in the coal gangue laser speckle image.

Four statistical characteristics are extracted based on the distribution of the gray histogram, namely gray mean (*m*), gray standard deviation (*σ*), skewness (*s*), and kurtosis (*k*). The calculation formulas are as follows:

(7) $$m=\sum_{b=0}^{255}bp\left(b\right)$$

(8) $$\sigma ={\left(\sum_{b=0}^{255}{\left(b-m\right)}^{2}p\left(b\right)\right)}^{1/2}$$

(9) $$s=\frac{{\sum_{b=0}^{255}\left(b-m\right)}^{3}p\left(b\right)}{\sigma^{3}}$$

(10) $$k=\frac{{\sum_{b=0}^{255}\left(b-m\right)}^{4}p\left(b\right)}{\sigma^{4}}-3$$

#### 2.2.2. Texture Features

The gray level co-occurrence matrix (GLCM) is defined by the joint probability density of pixels in two positions, which not only reflects the brightness distribution, but also the position distribution between pixels with the same brightness in the reaction space. In this paper, four independent texture features based on GLCM are extracted, including angular second moment (*ASM*), entropy (*ENT*), contrast (*CON*), and inverse difference moment (*IDM*).

*ASM* reflects the uniformity of an image’s gray distribution and texture fineness. A higher value indicates a more uniform and rough texture in the image. In the identification of coal and gangue, their roughness can be differentiated by comparing the second-order moments of angles between them. The calculation is as in Equation (11):

(11) $$ASM=\sum_{j=0}^{255}\sum_{k=0}^{255}{\left(P\left(j,k|d,\alpha \right)\right)}^{2}$$

*ENT*, reflecting the randomness of image information, is defined as Equation (12) when all values in the co-occurrence matrix are equal or pixel values exhibit maximum randomness. By comparing the entropy of coal and gangue images, their textural complexity can be differentiated.

(12) $$ENT=\sum_{j=0}^{255}\sum_{k=0}^{255}\left(P\left(j,k|d,\alpha \right)log_{2}P\left(j,k|d,\alpha \right)\right)$$

*CON*, reflecting the clarity of the image and the depth of the texture, is defined as Equation (13). In coal gangue identification, *CON* can reflect the clarity of mineral textures.

(13) $$\;CON=\sum_{j=0}^{255}\sum_{k=0}^{255}\left({\left(j-k\right)}^{2}P\left(j,k|d,\alpha \right)\right)$$

*IDM*, reflecting the degree of local change of image texture, is defined as Equation (14).

(14) $$\;IDM=\sum_{j=0}^{255}\sum_{k=0}^{255}\left(\frac{P\left(j,k|d,\alpha \right)}{1+{\left(j-k\right)}^{2}}\right)$$

where the matrix P(j,k|d,α) presents the number of pixel pairs (*j*, *k*) with adjacent intervals in the α direction. In this experiment, *α* is 45°, *d* is 1.

## 3. Experiment

### 3.1. Experimental Instrument

**Figure 4:**
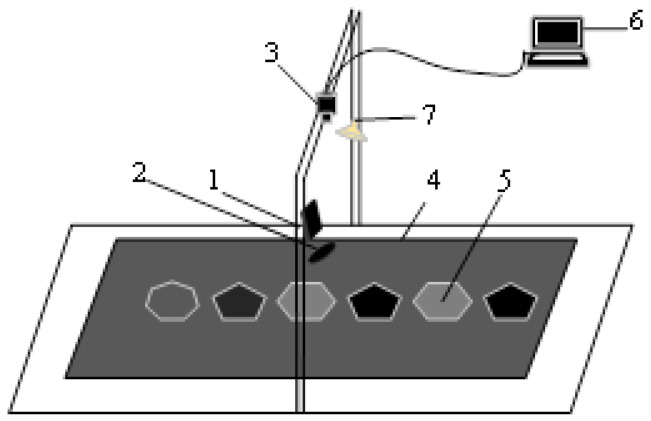
Coal gangue laser speckle image acquisition system. 1—Semiconductor laser; 2—collimating and beam expanding system; 3—industrial camera; 4—carrier platform; 5—coal and gangue samples; 6—computer; 7—adjustable LED light source.

A coal gangue laser speckle observation instrument was set up, as depicted in Figure 4. The instrument consists of five main parts: a semiconductor laser, the collimating and beam expanding system, an industrial camera, a carrier platform, a computer, and an adjustable LED light source. The semiconductor laser source (LSR638NL) with a wavelength of 638 nm and a power of 15.5 mW was used to provide a stable coherent light. This laser source emits light at a specific wavelength suitable for the scattering experiments. To focus and direct the laser beam onto the sample surface, a collimating and beam expanding system (GCO-2503) was employed. This system allows for continuous expansion of the spot diameter on the surface of the coal gangue samples up to 36 mm. It ensures proper illumination and collection of the scattered light for subsequent analysis. The scattered light was captured by an industrial camera (Aca2440-75uc) with a resolution of 2448 pixels × 2048 pixels. The captured light was then converted into a digital signal for further processing and analysis.

During the acquisition of mineral laser speckle images, the industrial camera was fixed on the carrier platform perpendicular to the surface of the coal gangue samples at 460 mm. To ensure a high contrast in the scattered speckle images of coal gangue, the exposure time of the industrial camera was set at 3000 μs. The laser source was positioned at an angle of 15° relative to the direction of the industrial camera, with the collimating and beam expanding system placed directly behind it. The coal gangue samples were placed on the carrier platform, and the coherent beam was irradiated onto the surface of the samples through the beam system. The industrial camera captured the laser speckle patterns with varying intensity distributions on the surface of the coal gangue samples. To control the illuminance environment, an adjustable LED light source was used to collect images under different lighting conditions.

### 3.2. The ROI Extraction of the Coal Gangue Laser Speckle Images

The background areas of the original coal gangue laser speckle images collected by the experiment are large. The speckle regions of interest were extracted from the laser speckle images in order to improve the speed and accuracy of the feature extraction of the coal gangue laser speckle images.

**Figure 5:**
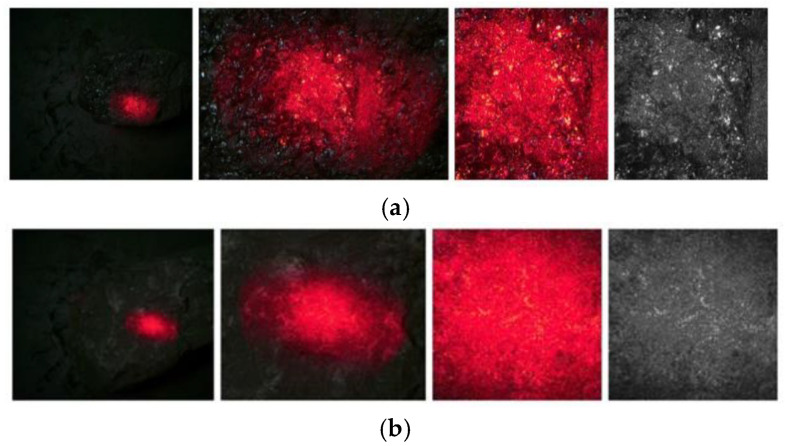
Extraction process of speckle ROI. (**a**) Coal; (**b**) gangue.

The propagation of coherent beams in the air is affected by suspended air particles, which introduce shot noise. In this study, Gaussian filtering was used to remove such noise from the laser speckle images during the acquisition process. The Gaussian filter convolves the image with a Gaussian kernel, which assigns weights to neighboring pixels based on their distance from the center pixel. These weights were determined using a Gaussian distribution with a specified standard deviation. By adjusting the standard deviation, we can fine-tune the denoising effect to match the specific noise characteristics and desired image quality. The Gaussian filtering method effectively balanced noise reduction and preservation of important image details, resulting in improved quality of the collected laser speckle images. After graying and Gaussian filtering, the maximum between-class variance (Otsu) [42] adaptive threshold algorithm was used to segment the target area of the coal gangue laser speckle. We extracted a region of interest (ROI) of size 250 × 250 pixels by center clipping. The process of speckle region of interest extraction is illustrated in Figure 5.

## 4. Results and Discussions

### 4.1. Feature Analysis of Coal Gangue Laser Speckle Images

In the experiment, 120 laser speckle images of coal and gangue under natural illuminance were collected, and their gray and texture features were extracted according to Section 2.2.1 and Section 2.2.2. To assess the effectiveness of different features in distinguishing between coal and gangue, we analyzed the inter-class separableness and intra-class compactness of these features. Inter-class separableness measures the level of differentiation between different classes, indicating that higher differences in features among different classes result in greater inter-class separableness. Intra-class compactness represents the similarity of samples within the same class, indicating that higher similarity among samples within the same class leads to greater intra-class compactness. We generated boxplots to analyze these laser speckle image features of coal gangue. The advantage of using boxplots is that they provide a visual means to better illustrate the spatial distribution of these features.

**Figure 6:**
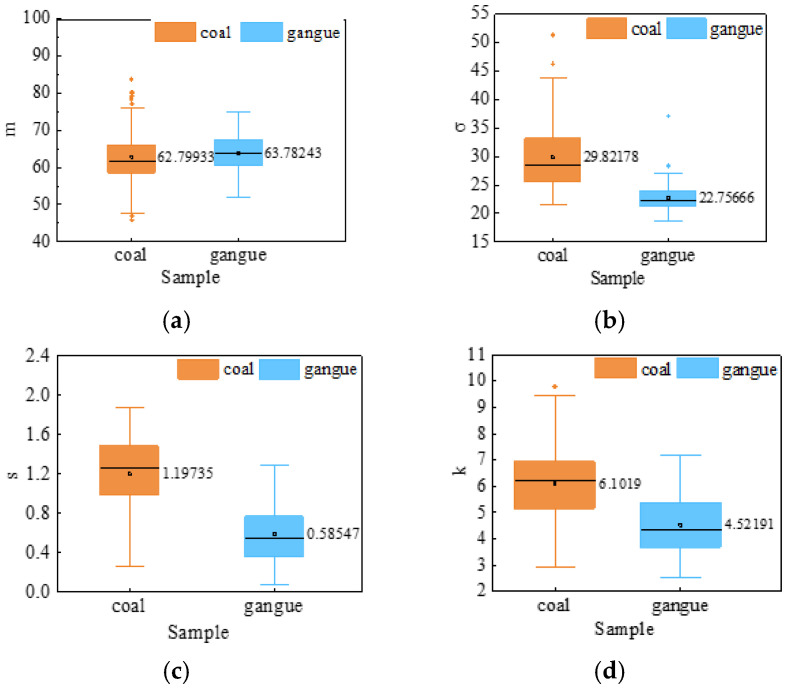
The box diagram of gray features. (**a**) m; (**b**) *σ*; (**c**) s; (**d**) k.

The boxplots of the four gray feature parameters of the coal gangue laser speckle images are shown in Figure 6. Figure 6a shows that the gray mean values of coal and gangue are roughly the same. The reason is that the gray distribution of the images was basically the same after the coal and gangue were irradiated by laser. As shown in Figure 6b, the gray standard deviation distribution of coal is more dispersed than that of gangue, and the average level is higher than that of gangue. Due to the large surface roughness of coal, the surface flares of speckle images are more distributed and more unevenly distributed, and the gray dispersion is large. As shown in Figure 6c, the average gray deviation of coal is higher than that of gangue, and the speckle distribution of gangue laser speckle images is smoother and more uniform. As shown in Figure 6d, the average gray kurtosis of coal is higher than that of gangue, but some data ranges overlap, indicating that the gray distribution of coal gangue laser speckle images is concentrated near the average value, but the dispersion of coal kurtosis is large, and the dispersion of the gray data distribution is large. Some feature components have individual abnormal values in the boxplots, such as the gray mean of coal, and the rationality of their existence is not ruled out without knowing the reasons for the abnormal values. This is also applicable to the following texture feature.

**Figure 7:**
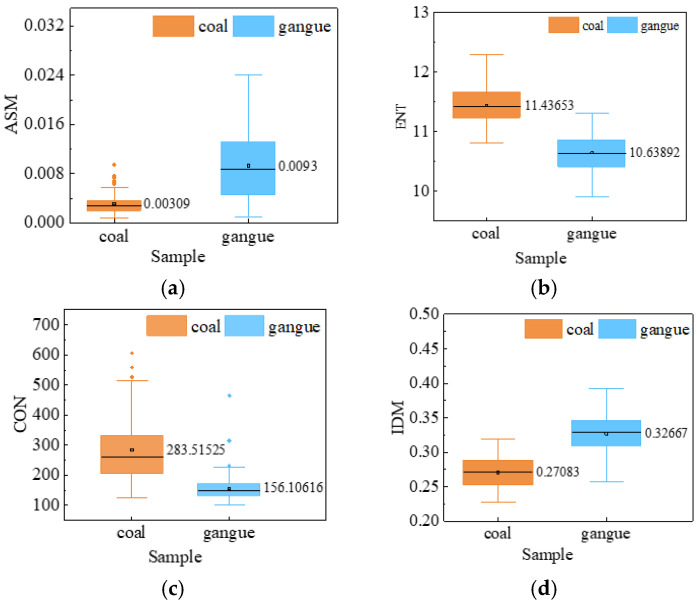
The box diagram of texture features. (**a**) ASM; (**b**) ENT; (**c**) CON; (**d**) IDM.

The box diagram of the four texture feature parameters of the coal gangue laser speckle images is shown in Figure 7. As shown in Figure 7a, the average value of the angular second moment of gangue is higher than that of coal, which reflects that the scattering degree of coal and gangue to a laser is different, and the difference between the samples’ angular second moment feature classes is large. After laser speckle irradiation, there were regular speckle distributions on the surface of gangue, and the large value of the angular second moment reflects the regular texture change of the gangue surface. The angular second moment value of the coal laser speckle image is low, and the distribution range is small. After laser speckle irradiation, an obvious uneven distribution of bright spots appears on the coal, the local gray change on the surface is obvious, the speckle distribution is dispersed, and the texture change is irregular. As shown in Figure 7b, the laser speckle image entropy of coal is large, the laser speckle image entropy of gangue is small, and the distribution range of the two is stable. The bright spots on the coal surface are randomly distributed. After laser irradiation, the randomly distributed high bright spots will appear on the speckle image, so the surface of the coal block is more complex, and the surface of the gangue is simpler [43]. As shown in Figure 7c, the average contrast of coal is higher than that of gangue, and the data dispersion is high, because the surface gully of coal is deeper than that of gangue, and the surface contour of different coal blocks has strong laser scattering, which enhances the contrast of the laser speckle. As shown in Figure 7d, the inverse difference moment value of the laser speckle images of gangue is larger, and the local change in gangue surface is slower. After laser irradiation, the images’ speckle distribution was more regular, and the speckle change rule was strong. However, the inverse difference moment of the coal laser speckle image is small, the gray change is uneven in different regions, and the local change of the surface is more irregular. 

**Figure 8:**
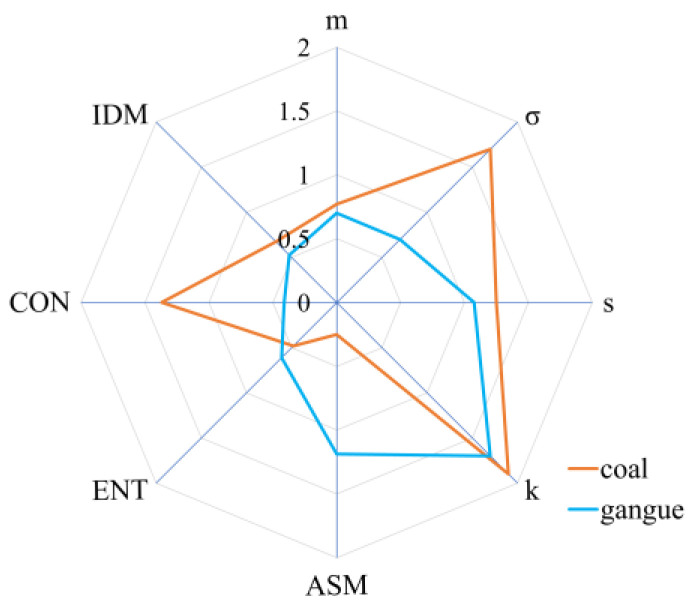
The standardized interquartile ranges of coal gangue laser speckle image features.

The median lines of coal laser speckle image features (except for gray mean) are observed to lie outside the box of corresponding gangue features. This observation suggests a significant difference in the central tendencies of these features between coal and gangue samples. Such findings provide evidence for the strong inter-class separability of the two categories based on the analyzed features. Furthermore, the texture features outperformed the gray features, as evidenced by the more pronounced spacing between the feature boxes of the two minerals. Moreover, the box lengths representing the interquartile ranges can indicate the degree to which a feature distribution is stretched or compressed. Since the image features vary significantly in magnitude, we conducted scale standardization on the interquartile ranges to facilitate the comparative analysis of all features in capturing the intra-class compactness of minerals. The standardized outcomes are depicted in Figure 8. The gray mean, gray deviation, angular second moment, entropy, and inverse difference moment demonstrate smaller scale ranges, indicating their significant capability in reflecting the intra-class compactness of minerals. Conversely, gray kurtosis exhibits the largest scale range, implying a weaker representation of internal similarities of the two mineral samples. Additionally, the blue curve encompasses a smaller area, indicating that the extracted laser speckle image features possess a superior ability to reflect the internal similarity within the gangue samples compared to the coal samples.

### 4.2. Verification of Coal Gangue Recognition Based on Laser Speckle Image

Using the aforementioned features of coal gangue laser speckle images as input, coal gangue recognition was realized based on the SVM recognition model. SVM [44,45], which constructs an optimal hyper-plane in high-dimensional or infinite-dimensional space, is a supervised learning model that improves the generalization ability of a learning machine by minimalizing structural risk and empirical risk while maximizing the confidence range. This allows for the derivation of effective statistical rules even with limited statistical samples. SVM simplifies classification and regression problems, particularly in small-sample scenarios. In this study, the SVM classifier is employed to categorize the 240 coal gangue laser speckle images collected from the aforementioned experiments. The classification is based on features derived from the gray-level co-occurrence matrix. For the SVM classifier, a linear kernel function was used, and the penalty factor C was set to 1. The database was divided into training sets (comprising 168 samples) and test sets (comprising 72 samples). The recognition accuracy of the coal gangue laser speckle images is presented in Table 1. 

Table 1 illustrates significant variations in the accuracy of SVM recognition models constructed using individual features. Specifically, the ENT (entropy) and IDM (inverse difference moment) features demonstrate higher accuracy rates of 93.1% and 87.5%, respectively. These findings underscore the importance of entropy and energy features in the recognition of coal gangue images. Conversely, the m (mean) and k (kurtosis) exhibit lower accuracy rates of 52.8% and 68.6%, respectively. This can be attributed to the weak performance of the m feature in terms of inter-class separability and the k feature in terms of intra-class compactness. Overall, the texture features of laser speckle images outperformed the grayscale features, with the former exhibiting an average accuracy rate 14.8% higher than the latter. By utilizing the fusion of all features extracted from coal and gangue laser speckle images, the SVM recognition model achieved a recognition accuracy of 94.4%, surpassing the accuracy obtained by using any individual feature alone. These results highlight the complementary information provided by different features and the improved classification results achieved through feature fusion.

### 4.3. Identification Stability of Coal Gangue Laser Speckle Images

**Figure 9:**
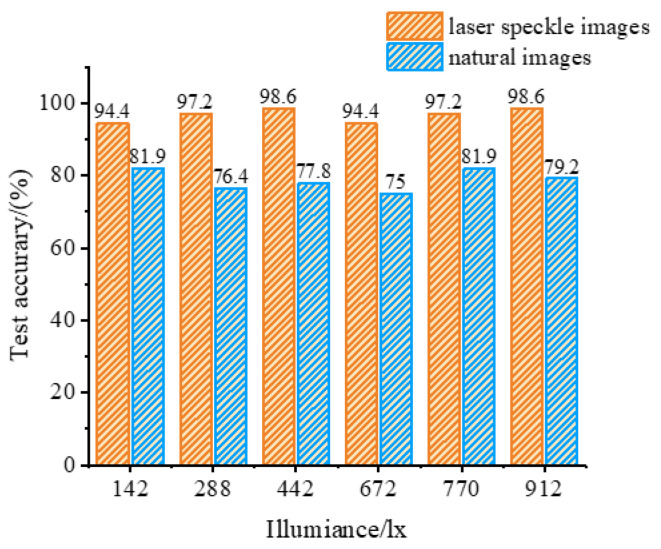
The recognition accuracy with different illuminances.

The identification feasibility of coal gangue laser speckle images was explored in this study. However, only one kind of illuminance was studied, whereas the illuminance environment of the actual coal caving site is weak and unstable, which seriously affects the feature extraction and coal gangue recognition accuracy. Therefore, in order to verify the role of the research method in this paper to solve this problem, in the future, it is necessary to collect more kinds of illuminance from different minerals to assess whether the proposed research method is universal. In order to further discuss and verify the stability of coal gangue laser speckle image recognition to illuminance, an image acquisition system with controllable illuminance was established to collect the coal gangue natural images and coal gangue laser speckle images, and each group was illuminated with an illuminance of 100, 288, 442, 672, 770, and 912 lx. The gray and texture features of the coal gangue natural images and coal gangue laser speckle images under different illuminances were abstracted, and the training set and test set were divided according to 7:3; the recognition accuracy of two different images with different illuminances is shown in Figure 9.

It can be observed from Figure 9 that the accuracy of coal gangue laser speckle image recognition based on gray features and texture features remains relatively stable under different lighting conditions. The average accuracy of coal gangue laser speckle image recognition under various lighting conditions is 96.7%, with a standard deviation of the recognition accuracy of 1.7%. In comparison, the average accuracy of coal gangue recognition based on natural images is 78.7%, with a standard deviation of the recognition accuracy of 2.6%, and the highest recognition rate achieved is 81.9%. This indicates that natural images exhibit larger fluctuations in recognition accuracy with changes in lighting conditions. The experimental results demonstrate that the proposed coal gangue recognition method based on laser speckle images achieves higher and more stable recognition accuracy under varying lighting conditions to a certain extent.

## 5. Conclusions

This paper presents a novel method for identifying coal and gangue under different lighting conditions using laser speckle imaging technology. The analysis of four gray features and four texture features confirms that laser speckle images effectively demonstrate the differences in the distribution of these minerals. The extracted texture features show better inter-class separability and intra-class compactness compared to the gray features. Using a single texture feature, the SVM model achieves an average recognition accuracy of 86.1% for coal and gangue, outperforming the accuracy of 71.3% achieved by a single gray feature. By fusing all the extracted features, the model’s recognition capability is positively optimized, resulting in an increased accuracy of 94.4%. Furthermore, the proposed method achieves an average recognition accuracy of 96.7% under different lighting conditions, with a maximum accuracy of 98.7%. These results significantly surpass the recognition accuracy obtained from natural coal and gangue images using the same features. The method also exhibits good stability in coal and gangue recognition with a smaller standard deviation of recognition accuracy. 

Based on the above aspects, the proposed method shows great promise for coal and gangue identification in practical production applications, particularly under challenging lighting conditions. It can also be extended to address other image-related tasks in coal mining processes conducted in low-light and unstable environments. For instance, it can be employed for identifying equipment failures in underground mines and detecting and measuring deformations in roadway structures. Furthermore, this paper only focuses on training models using the eight typical coal and gangue image features. Future research can explore feature design methods to enhance the performance of the proposed method. For example, researchers can incorporate morphological features to further explore the performance of local texture information in laser speckle for reflecting mineral categories. Additionally, considering the impact of model parameters and structure on recognition accuracy, research on model optimization methods is necessary to improve their recognition accuracy and stability.

## Figures and Tables

**Table 1:** Recognition accuracy of coal gangue laser speckle images.

Feature	m	σ	s	k	ASM	ENT	CON	IDM	Feature Fusion
Accuracy/%	52.8	83.3	80.6	68.6	80.6	93.1	83.3	87.5	94.4

## Data Availability

Data are contained within the article.
